# Active trachoma prevalence among Yemeni children in previously highly endemic districts following three rounds of mass drug administration: progress toward eliminating trachoma as a public health problem

**DOI:** 10.1186/s12889-026-29095-1

**Published:** 2026-08-19

**Authors:** Sami A. Al-Haidari, Yahia A. Raja’a, Mohammed A. K. Mahdy, Talal A. Haider, Rashad Abdul-Ghani

**Affiliations:** 1 Department of Community Medicine, Faculty of Medicine and Health Sciences, Sana’a University, Sana’a, Yemen; 2 Department of Medical Parasitology, Faculty of Medicine and Health Sciences, Sana’a University, Sana’a, Yemen; 3 Department of Ophthalmology, Faculty of Medicine and Health Sciences, Sana’a University, Sana’a, Yemen; 4 Tropical Disease Research Center, Faculty of Medicine and Health Sciences, University of Science and Technology (USTY), Sana’a, Yemen

**Keywords:** Trachoma, Children, Mass drug administration, Yemen

## Abstract

**Background:**

This study estimated the prevalence of trachomatous inflammation–follicular among children aged 1–9 years (TF_1–9_) in previously highly endemic districts of Yemen following three rounds of mass drug administration (MDA) and identified its predictors.

**Methods:**

A household-based cross-sectional study using a two-stage cluster sampling approach was conducted among 1290 children from randomly selected households across six districts within evaluation units (EUs) with pooled 2013 baseline TF_1–9_ prevalence exceeding 10% in Ibb and Hodeidah governorates. These districts received three rounds of MDA in 2021, 2023, and 2024. A structured questionnaire was used to collect data on children’s sociodemographic characteristics and household water, sanitation, and hygiene conditions. The children’s eyes were examined for trachoma using the World Health Organization (WHO) simplified trachoma grading system. Univariate analysis was performed to assess the associations between independent variables and TF_1–9_, and multivariable binary logistic regression was used to identify predictors of TF_1–9_ at a statistical significance level of *P* < 0.05.

**Results:**

TF_1–9_ was prevalent among 0.9% (11/1290) of children in previously highly endemic districts of Ibb and Hodeidah governorates, with reductions ranging from 83.5% to 95.2% compared with pooled 2013 EU baselines. Multivariable binary logistic regression analysis showed that households lacking improved sanitation facilities (AOR = 10.2, 95% CI: 1.70–61.19; *P* = 0.011) and the presence of nasal and/or ocular discharge on a child’s face (AOR = 60.0, 95% CI: 6.16–584.54; *P* < 0.001) were independent predictors of TF^1–9^. However, because only 11 TF-positive children were observed, the resulting CIs were wide, indicating substantial imprecision and potential instability of these estimates.

**Conclusions:**

The decline in TF_1–9_ prevalence to below the WHO threshold of 5% in previously highly endemic districts of Ibb and Hodeidah governorates suggests that substantial progress has been made toward achieving the active trachoma component of the elimination criteria and that further MDA is not currently required in these districts. Nevertheless, post-MDA surveillance surveys should be conducted to detect any potential recrudescence. Poor household sanitation and facial uncleanliness with nasal and/or ocular discharge may independently predict TF_1–9_, although these associations should be interpreted with caution because of the associated uncertainty. Improving access to adequate sanitation and promoting facial cleanliness remain key strategies for reducing the risk of trachoma recrudescence following MDA cessation.

## Background

Trachoma, a bacterial eye infection caused by *Chlamydia trachomatis*, is a leading preventable cause of visual impairment and blindness worldwide [1]. It ranks as the second most common cause of blindness in Africa, Asia, and the Middle East [2] and is classified by the World Health Organization (WHO) as a neglected tropical disease (NTD) [3]. Transmission occurs primarily through contact with ocular or nasal secretions from infected individuals, contaminated hands or objects, or by eye-seeking flies [4]. Overcrowding, inadequate sanitation, and limited access to clean water facilitate transmission. Repeated conjunctival infections can cause corneal scarring and inward turning of the eyelid, leading to trachomatous trichiasis (TT) [1]. If left untreated, TT can cause corneal opacity and potentially irreversible blindness [1].

In 2024, WHO estimated that 1.9 million people were living with moderate-to-severe visual impairment or blindness attributable to trachoma across 29 countries [1]. Moreover, about 92 million people were living in trachoma-endemic areas and remained at risk of blindness [1]. The WHO-endorsed SAFE strategy—Surgery (S), Antibiotics (A), Facial cleanliness (F), and Environmental improvement (E)—combines tertiary prevention through eyelid surgery with annual antibiotic mass drug administration (MDA) in areas where TF prevalence is 5% or higher, alongside primary prevention measures that promote facial cleanliness and improved environmental sanitation [5]. Under the WHO framework for eliminating trachoma as a public health problem, the prevalence of TT among individuals aged 15 years and older should be reduced to below 0.2% and that of trachomatous inflammation–follicular (TF) in children aged 1–9 years (TF_1–9_) should fall below 5% [6]. Significant progress has been made in the Eastern Mediterranean Region toward eliminating trachoma as a public health problem, with Oman, Morocco, and Iran having reached this milestone [6]. In Yemen, trachoma remains a major public health problem. However, there has been growing interest in trachoma control over the past few years in response to its substantial public health burden in the country [7].

Baseline mapping conducted in Yemen from 2013 to 2015 identified evaluation units (EUs) requiring MDA, including those in highly endemic areas of Ibb and Hodeidah governorates [8, 9]. Subsequent surveys documented active trachoma in parts of these governorates and in Sana’a [10–12]. Three rounds of azithromycin MDA were implemented in selected highly affected districts of Ibb and Hodeidah in 2021, 2023, and 2024. However, updated prevalence data are needed to guide decisions regarding MDA cessation, post-MDA surveillance, and continued implementation of the F and E components of the SAFE strategy. Therefore, this study aimed to estimate TF_1–9_ prevalence in previously highly endemic districts of Ibb and Hodeidah governorates following the three MDA rounds, compare the 2024 estimates with the available pooled 2013 EU-level baseline estimates, and assess sociodemographic and water, sanitation, and hygiene (WASH) factors potentially associated with residual TF_1–9_. These findings can have direct programmatic implications for Yemen’s National Trachoma Elimination Programme, supporting decisions on MDA cessation, the prioritization of post-MDA surveillance, and the continued implementation of the facial cleanliness and environmental improvement components of the SAFE strategy.

## Methods

### Study design, population and setting

**Fig. 1:**
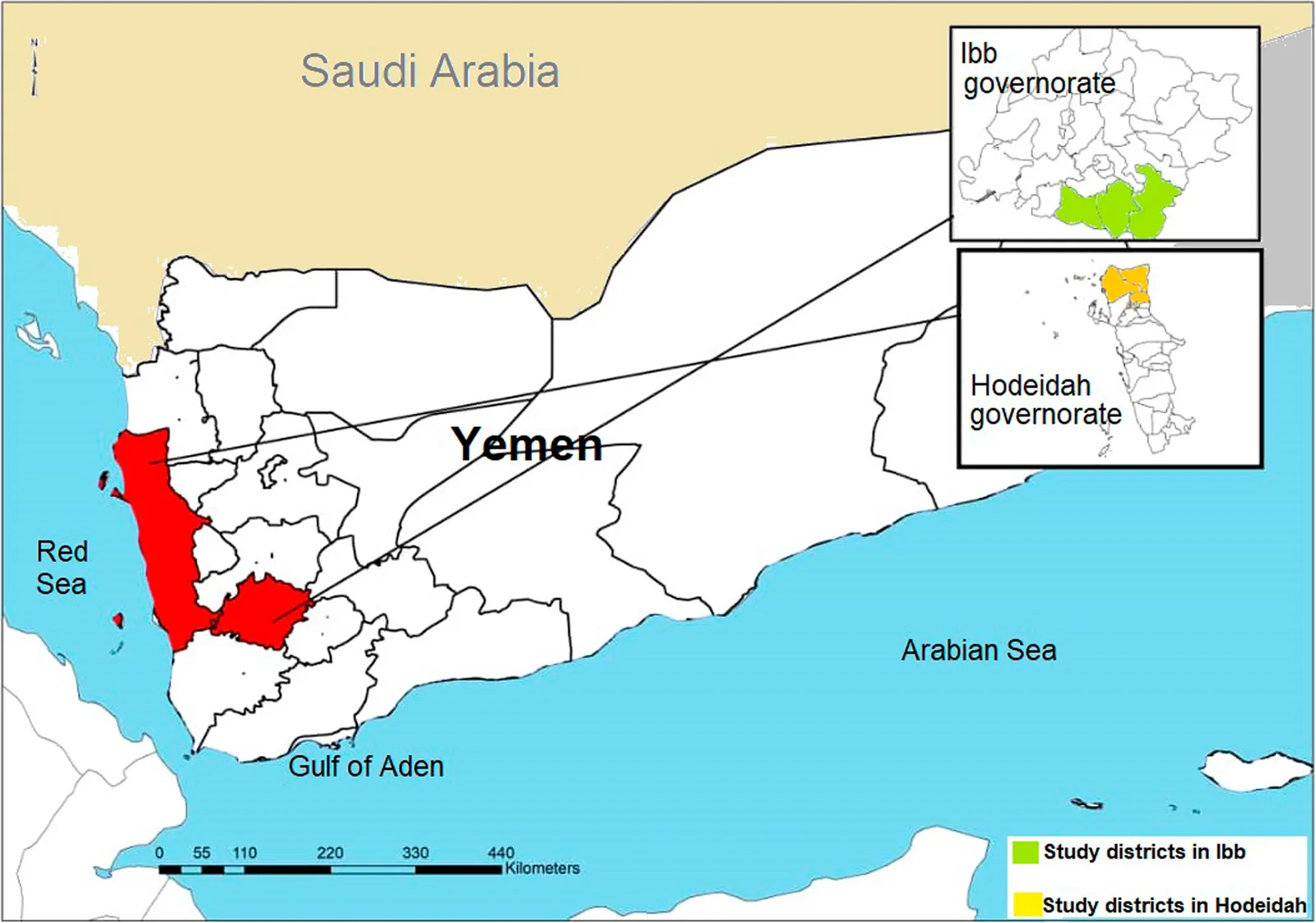
Map of Yemen showing the location of the survey districts in Ibb and Hodeidah governorates

A household-based cross-sectional study was conducted among children aged 1 to 9 years from six randomly selected districts in September 2024. These districts were eligible for selection based on the following criteria: documented high endemicity for trachoma in the 2013 baseline survey, with TF prevalence exceeding 10% in their EUs; implementation of three rounds of azithromycin MDA in 2021, 2023, and 2024; and average programmatic MDA coverage exceeding the WHO-recommended threshold of 80% across the three rounds. The selected districts were As Sabrah, As Sayyani, and Dhi As Sufal in Ibb governorate and Al Qanawis, Alluheyah, and Az Zuhrah in Hodeidah governorate (Fig. 1). Children of either sex were eligible if they were permanent residents of the selected districts and informed consent was provided by the household head or a legal representative.

### Sample size calculation

Using the WHO-recommended design parameters for population-based trachoma surveys [13], a sample size of 970 children was calculated, assuming an expected TF prevalence of 4%, a 95% confidence level, an absolute precision of 2%, and a design effect of 2.63. The sample size was subsequently increased to 1290 using an inflation factor of 1.33.

### Sampling strategy

Consistent with the 2013 survey, a two-stage cluster sampling approach was employed. In the first stage, 30 clusters were selected using systematic random sampling. The study districts were divided into clusters based on the WHO definition of EUs for trachoma surveys [13]. Villages were the main sampling units, and households were the secondary sampling units. In the second stage, a compact segment sampling method was used to select households from the lists within these clusters, with the number of households proportional to each cluster’s population size. A household was defined as all individuals residing in a dwelling unit as their regular residence, regardless of whether the individuals were in single or multiple marriages. The lists of households with eligible children were obtained from the Neglected Tropical Disease Programme of the Ministry of Health and Environment. Households were selected from these lists by simple random sampling.

The final sample comprised 602 (46.7%) children recruited from Ibb governorate (129 from As Sabrah, 172 from As Sayyani, and 301 from Dhi As Sufal), while 688 children (53.3%) were recruited from Hodeidah governorate (172 from Al Qanawis, 215 from Alluheyah, and 301 from Az Zuhrah). The heads of the selected households, or their adult representatives, were informed about the purpose of the study, and their consent for voluntary participation was obtained. If more than one eligible child was present within a selected household, one child was selected by simple random sampling. Because field records did not consistently document households with absent children or refusals by household heads, replacement households, or reasons for non-participation, the household-level response rate could not be calculated reliably.

### Data collection and trachoma examination

The household head was interviewed using a structured questionnaire to collect the child’s sociodemographic characteristics and assess the availability and status of WASH conditions. The questionnaire was developed after reviewing the relevant literature in Yemen and comparable trachoma-endemic settings [10–12, 14–16]. It was developed in Arabic and reviewed by the study team to ensure clarity, cultural appropriateness, and consistency with the intended concepts. Before data collection, the questionnaire was pilot-tested with 20 households in a neighboring district not included in the final analysis. Based on the pilot test findings, minor modifications were made to the wording and sequencing of certain questions to enhance comprehension and interview flow, but no study variables or response categories were altered. Household interviews were conducted by trained field enumerators who were independent of the clinical assessment team.

After the household interview, ophthalmologists assessed facial cleanliness by direct visual observation before examining children’s eyes. Facial cleanliness was recorded as a binary variable based on the presence or absence of visible nasal and/or ocular discharge. A child’s face was classified as unclean if visible discharge was present at the time of examination and as clean if no such discharge was observed. This assessment was based on a standardized field definition used by the survey team, although photographic grading was not performed. The ophthalmologists performing clinical examinations did not administer the questionnaire, have access to the completed questionnaires, or use the collected sociodemographic and WASH information during facial cleanliness assessment or TF grading. Nevertheless, because no formal masking procedure was implemented, the examiners may have been aware of some household characteristics observed during fieldwork.

The eyes of children were examined for TF using the WHO trachoma grading system [9, 17, 18] by qualified ophthalmologists with prior experience in national trachoma surveys. The ophthalmologists completed standardized training and certification in accordance with WHO and International Trachoma Initiative (ITI) guidelines. Training included an initial theoretical review of the WHO trachoma grading system, followed by practical sessions using WHO-recommended image sets for TF diagnosis. Certification was granted only after examiners achieved a minimum inter-rater agreement, expressed as a kappa statistic of at least 0.80, compared with an expert gold-standard grader. During the survey, random inter-observer consistency checks were performed in the field to maintain diagnostic accuracy and minimize potential misclassification, which was particularly important given the low TF_1–9_ prevalence observed.

TF was defined as the presence of five or more follicles, each measuring at least 0.5 mm in diameter, in the central part of the upper tarsal conjunctiva [17]. To prevent cross-infection among participants, the investigators wore disposable gloves or sanitized their hands with 70% alcohol after each examination. Children diagnosed with TF received immediate treatment, including two tubes of 1% tetracycline eye ointment and azithromycin tablets at no cost, and were referred to the nearest primary healthcare center for monitoring and follow-up. Their household contacts were also screened and treated in accordance with national trachoma control guidelines. Children and household contacts with suspected advanced ocular disease or complications were referred to the nearest secondary ophthalmic care facility.

### Data analysis

Data were initially entered into Microsoft Excel spreadsheets, checked for duplicates and inconsistencies, and then analyzed using IBM SPSS Statistics, version 26.0 (IBM Corp., Armonk, NY, USA), with statistical significance set at *P* < 0.05. Categorical data were presented as frequencies and percentages, whereas continuous variables were summarized using the mean and standard deviation (SD) when normally distributed or the median and interquartile range (IQR) when non-normally distributed. TF_1–9_ prevalence was calculated with its corresponding 95% confidence interval (CI).

Univariate analysis was performed to assess associations between each independent variable and TF_1–9_ using the chi-square test or Fisher’s exact test, as appropriate, and the odds ratios (ORs) of these associations with their corresponding CIs were reported. Variables with *P* < 0.25 in univariate analysis were considered candidates for inclusion in multivariable binary logistic regression model to identify independent predictors of TF_1–9_. Candidate variables were entered into the initial model, and adjusted ORs (AORs) and 95% CIs were calculated. Before fitting the multivariable binary logistic regression model, collinearity among candidate variables was assessed using tolerance, variance inflation factors (VIFs), and cross-tabulation with calculation of phi coefficients. Variables showing severe collinearity were not entered together into the final model to avoid unstable estimates. Severe collinearity was detected between “household latrine availability” and “cleanliness of the child’s face from nasal and/or ocular discharge” (tolerance = 0.039; VIF > 25; phi coefficient = 0.976, *P* < 0.001). No other combination of candidate variables showed evidence of severe collinearity, with tolerance values ranging from 0.944 to 0.993 and VIFs ranging from 1.007 to 1.060. Consequently, “household latrine availability” was excluded from the final model.

## Results

### Sociodemographic characteristics of the study population

Among the 1290 children enrolled in the study, more than half were males (54.7%) and aged ≤ 5 years (58.8%). The children had a mean age of 5.0 ± 2.4 years, whereas the mean age of their household heads was 40.5 ± 11.4 years. The median household size was 7 members (IQR: 4), and 49.2% of the children lived in households comprising more than 7 members. More than half of the heads of households for these children were females (54.4%), and 61.4% were aged ≤ 40 years. Most fathers were literate (65.6%), while most mothers were illiterate (79.4%). Farming was the most frequently reported paternal occupation (41.6%), while nearly one-third of the fathers were unemployed (Table 1).

**Table 1:** Characteristics of children enrolled in the study*

Characteristic	*n*	(%)
Sex of the child		
Male	706	(**54.7**)
Female	584	(**45.3**)
Age of the child (years)		
≤ 5	759	(**58.8**)
> 5	531	(**41.2**)
Mean ± SD: 5.0 ± 2.4		
Household size (members)		
≤ 7	655	(**50.8**)
> 7	635	(**49.2**)
Median (IQR): 7.0 (4)		
Sex of the household head		
Male	588	(**45.6**)
Female	702	(**54.4**)
Age of the household head (years)		
≤ 40	792	**(61.4)**
> 40	498	**(38.6)**
Mean ± SD (range): 40.5 ± 11.4 (21–90)		
Father’s literacy status		
Literate	846	**(65.6)**
Illiterate	444	**(34.4)**
Mother’s literacy status		
Literate	266	**(20.6)**
Illiterate	1024	**(79.4)**
Father’s occupation		
Farmer	537	**(41.6)**
Unemployed	374	**(29.0)**
Employed	192	**(14.9)**
Daily laborer	187	**(14.5)**

*The total number of children was 1290. *SD* Standard deviation, *IQR* Interquartile range

### Prevalence of TF_1–9_

The overall prevalence of TF_1–9_ in the previously highly endemic districts of Ibb and Hodeidah governorates was 0.9% (11/1290; 95% CI: 0.43–1.52). In Ibb, the overall prevalence of TF_1–9_ was 0.8% (5/602; 95% CI: 0.27–1.93), with rates of 1% (3/301) in Dhi As Sufal district, 0.8% (1/129) in As Sabrah and 0.6% (1/172) in As Sayyani. In Hodeidah, the overall prevalence was 0.9% (6/688; 95% CI: 0.32–1.89), with the highest rate observed in Al Qanawis district (1.7%; 3/172), followed by 0.7% (2/301) in Az Zuhrah and 0.5% (1/215) in Alluheyah (Table 2).

**Table 2:** Prevalence of TF_1–9_ following three rounds of MDA in previously highly endemic districts of Ibb and Hodeidah governorates, Yemen, 2024

Governorate	Districts	*N*	TF_1–9_ prevalence after three rounds of MDA *n* (%; 95% CI)
Ibb	As Sabrah	129	1 (0.8; 0.02–4.24)
	As Sayyani	172	1 (0.6; 0.01–3.20)
	Dhi As Sufal	301	3 (1.0; 0.21–2.88)
	**All districts**	**602**	**5 (0.8; 0.27–1.93)**
Hodeidah	Al Qanawis	172	3 (1.7; 0.39–5.01)
	Alluheyah	215	1 (0.5; 0.01–2.56)
	Az Zuhrah	301	2 (0.7; 0.08–2.38)
	**All districts**	**688**	**6 (0.9; 0.32–1.89)**
Overall prevalence		**1290**	**11 (0.9; 0.43–1.52)**

*N* Number of children examined, *n* Number of children with trachoma, *TF*_*1–9*_ Trachomatous inflammation–follicular among children aged 1–9 years, *MDA* Mass drug administration, *CI* Confidence interval

### Comparison of TF_1–9_ prevalence in 2024 following three MDA rounds with pooled 2013 EU baseline

Using the pooled 2013 EU baselines as comparators, TF_1–9_ prevalence declined by 93.7% across surveyed districts in Ibb and by 91.3% in Hodeidah, corresponding to an overall reduction of 92.2%. At the district level, the largest reduction was observed in As Sayyani (95.2%), followed by Alluheyah (95.1%), As Sabrah (93.7%), Az Zuhrah (93.2%), and Dhi As Sufal (92.1%), whereas the smallest reduction of 83.5% was observed in Al Qanawis (Table 3). Nevertheless, because the 2013 comparators were pooled EU-level estimates rather than district-specific baseline measurements, these percentages represent descriptive programmatic comparisons rather than district-specific longitudinal changes attributable solely to MDA.

**Table 3:** Comparison of 2024 TF_1–9_ prevalence with pooled 2013 EU baseline estimates in previously highly endemic districts of Ibb and Hodeidah governorates, Yemen

Governorate	Districts	TF_1–9_ prevalence (%)		Percentage Reduction
		2024 (following 3 MDA rounds)	2013 (baseline) ^a^	
Ibb	As Sabrah	0.8	12.6	93.7
	As Sayyani	0.6	12.6	95.2
	Dhi As Sufal	1.0	12.6	92.1
	**All districts**	**0.8**	**12.6**	**93.7**
Hodeidah	Al Qanawis	1.7	10.3	83.5
	Alluheyah	0.5	10.3	95.1
	Az Zuhrah	0.7	10.3	93.2
	**All districts**	**0.9**	**10.3**	**91.3**
Overall prevalence		**0.9**	**11.5**	**92.2**

*TF*_*1–9*_ Trachomatous inflammation–follicular among children aged 1–9 years, *MDA* Mass drug administration, *EU* Evaluation unit

^a^Pooled 2013 baseline estimates for the EU comprising the corresponding districts

### Association of sociodemographic characteristics with TF_1–9_

Children living in households headed by individuals aged > 40 years had significantly higher odds of TF_1–9_ than those living in households headed by individuals aged ≤ 40 years (OR = 7.3, 95% CI: 1.56–33.79; *P* = 0.004). However, no significant associations were found between TF_1–9_ and children’s sex or age, household size, the sex of the household head, or parental literacy or unemployment status (Table 4).

**Table 4:** Association of sociodemographic characteristics with TF_1–9_ in previously highly endemic districts of Ibb and Hodeidah governorates, Yemen, 2024

Characteristic	*N*	Presence of TF_1–9_					*P* value
		*n*	(%)	OR	(95% CI)		
Sex of the child							
Male	706	6	**(0.8)**	**1.0**	(0.30–3.27)		1.000
Female	584	5	**(0.9)**	Reference			
Age of the child (years)							
$\:\le\:$5	759	9	**(1.2)**	**3.2**	(0.68–14.75)		0.138
> 5	531	2	**(0.4)**	Reference			
Household size (members)							
$\:\le\:$7	655	3	**(0.5)**	Reference			
> 7	635	8	**(1.3)**	**2.8**		(0.73–10.50)	0.139
Sex of the household head							
Male	588	5	**(0.9)**	**1.0**		(0.31–3.31)	0.993
Female	702	6	**(0.9)**	Reference			
Age of the household head (years)							
$\:\le\:$40	792	2	**(0.3)**	Reference			
> 40	498	9	**(1.8)**	**7.3**	(1.56–33.79)		0.004
Father’s literacy status							
Literate	846	7	**(0.8)**	Reference			
Illiterate	444	4	**(0.9)**	**1.1**	(0.32–3.74)		1.000
Mother’s literacy status							
Literate	266	3	**(1.1)**	Reference			
Illiterate	1024	8	**(0.8)**	**0.7**	(0.18–2.62)		0.706
Father’s employment status							
Unemployed	374	4	**(1.1)**	Reference			
Employed/working ^a^	916	7	**(0.8)**	**0.7**	(0.21–2.4)		0.739

*N* Number examined, *n* Number positive, *TF*_*1–9*_ Trachomatous inflammation–follicular among children aged 1–9 years, *OR* Odds ratio, *CI* Confidence interval

^a^ These included employees, farmers and daily laborers

### Association of WASH characteristics with TF_1–9_

The absence of latrine facilities in the household (OR = 147.9, 95% CI: 18.70–1169.64; *P* < 0.001), unimproved sanitation facilities (OR = 23.7, 95% CI: 4.87–115.02; *P* < 0.001) and facial uncleanliness with nasal and/or ocular discharge (OR = 65.7, 95% CI: 13.96–309.00; *P* < 0.001) were significantly associated with TF_1–9_. In contrast, no significant associations were found between TF_1–9_ and the source of household water, the distance to the household water source, the source of household water used for washing faces, or the regular washing of hands and faces with water and soap (Table 5).

**Table 5:** Association of WASH characteristics with TF_1–9_ in previously highly endemic districts of Ibb and Hodeidah governorates, Yemen, 2024

Characteristic	*N*	Presence of TF_1–9_					*P* value
		*n*	(%)	OR	(95% CI)		
Availability of latrine facilities in the household							
Yes	1199	1	**(0.1)**	Reference			
No	91	10	**(11.0)**	**147.9**	(18.70–1169.64)		< 0.001
Source of household water							
Unimproved	800	6	**(0.8)**	**0.7**	(0.22–2.42)		0.757
Improved	490	5	**(1.0)**	Reference			
Distance to water source (minutes)							
> 30	348	6	**(1.7)**	**3.3**	(1.00–10.84)		0.079
≤ 30	942	5	**(0.5)**	Reference			
Source of household water used for washing faces							
Unimproved	800	5	**(0.6)**	**0.5**	(0.15–1.67)		0.350
Improved	490	6	**(1.2)**	Reference			
Sanitation facilities in the household ^a^							
Unimproved	160	7	**(4.4)**	**23.7**	(4.87–115.02)		< 0.001
Improved	1037	2	**(0.2)**	Reference			
Self-reported regular washing of hands and faces with water and soap							
No	644	8	**(1.2)**	**2.7**		(0.71–10.21)	0.129
Yes	646	3	**(0.5)**	Reference			
Cleanliness of a child’s face from nasal and/or ocular discharge							
No	91	9	**(9.9)**	**65.7**		(13.96–309.00)	< 0.001
Yes	1199	2	**(0.2)**	Reference			

*N* Number examined, *n* Number positive, *TF*_*1–9*_ Trachomatous inflammation–follicular among children aged 1–9 years, *WASH* Water, sanitation and hygiene, *OR* Odds ratio, *CI* Confidence interval

^a^93 missing cases (91 children from households without latrine facilities were not applicable, and 2 children from households with latrines had missing sanitation facility type)

### Independent predictors of TF_1–9_

Multivariable binary logistic regression analysis showed that unimproved household sanitation facilities (AOR = 10.2, 95% CI: 1.70–61.19, *P* = 0.011) and the presence of nasal and/or ocular discharge on a child’s face (AOR = 60.0, 95% CI: 6.16–584.54, *P* < 0.001) were independent predictors of TF_1–9_ (Table 6). However, because only 11 TF-positive cases were observed, the resulting 95% CIs were wide, suggesting substantial imprecision and potential instability in the adjusted estimates. Therefore, these estimates should be interpreted as exploratory and requiring confirmation in larger studies, rather than as precise measures of independent associations.

**Table 6:** Independent predictors of TF_1–9_ in previously highly endemic districts of Ibb and Hodeidah governorates, Yemen, 2024

Predictor ^a^	AOR	(95% CI)	*P* value
Unimproved household sanitation facilities	**10.2**	(1.70–61.19)	0.011
Presence of nasal and/or ocular discharge on a child’s face	**60.0**	(6.16–584.54)	< 0.001

*TF*_*1–9*_ Trachomatous inflammation–follicular among children aged 1–9 years, *AOR* Adjusted odds ratio, *CI* Confidence interval

^a^ These estimates are exploratory and should be interpreted with caution because the model was based on only 11 TF-positive cases

## Discussion

The present study found that the three rounds of azithromycin MDA implemented over the period 2021–2024 were followed by a marked reduction in the prevalence of TF_1–9_ across the previously highly endemic districts of Ibb and Hodeidah. Overall, the prevalence of TF_1–9_ declined from 11.5% in 2013 to 0.9% in 2024, representing a 92.2% reduction. When the 2024 district-level prevalence estimates were compared with the corresponding pooled 2013 EU baselines, the reductions exceeded 90% in all districts except Al Qanawis, where the reduction was estimated at 83.5%. However, these comparisons should be interpreted with caution because the 2013 estimates were pooled at the EU level and district-specific baseline estimates were unavailable. Although the observed pattern is consistent with MDA having contributed substantially to the decline in TF_1–9_ prevalence, the cross-sectional design precludes attributing the reduction solely to MDA. Other factors, including secular trends, population movement, access to healthcare, and unmeasured changes in WASH conditions, may also have contributed. The comparatively higher prevalence estimate of 1.7% and the smaller estimated reduction of 83% in Al Qanawis also warrant cautious interpretation. This prevalence estimate was based on only three TF-positive children, and its 95% CI overlapped with those of the other districts. Furthermore, the 10.3% comparator represented a pooled EU-level baseline rather than a district-specific estimate. The apparently smaller reduction may therefore reflect sampling variation or a mismatch between the pooled baseline and the true district-level prevalence, rather than a true difference in programme performance. If subsequent surveillance confirms residual transmission, plausible district-level contributors may include incomplete participation in repeated MDA rounds, persistent deficiencies in water, sanitation, and hygiene, limited access to healthcare services, and conflict-related population movement or displacement. However, these explanations remain speculative because district-specific data on MDA participation, longitudinal WASH conditions, healthcare access, and population mobility were unavailable. Accordingly, Al Qanawis should be prioritized for enhanced surveillance and targeted programmatic assessment. The first MDA round in 2021 also coincided with the coronavirus disease 2019 (COVID-19) pandemic, which may have affected drug-distribution logistics, community participation, access to routine health services, and household hygiene practices. However, COVID-related disruptions were not systematically documented, and their potential influence on MDA implementation and the observed reduction in TF_1–9_ prevalence could not be assessed. The approximately two-year interval between the first and second MDA rounds deviated from the WHO-recommended annual MDA schedule for districts with TF_1–9_ prevalence above 10% [19]. Evidence synthesized from azithromycin MDA studies indicates that annual treatment may reduce ocular *C. trachomatis* infection more effectively than biennial treatment in some settings, although the effects on clinical TF have been inconsistent [20]. Moreover, a cluster-randomized trial found that annual and twice-yearly MDA achieved comparable infection prevalence at 42 months; however, twice-yearly treatment led to elimination approximately 7.5 months earlier [21]. Interruptions in MDA may permit transmission and re-emergence of infection [22]. Accordingly, the two-year interval between treatment rounds in the present study may have allowed transmission to persist and delayed the cumulative effect of MDA. Although the low prevalence of TF_1–9_ in 2024 suggests substantial programmatic progress, the independent effect of the treatment gap cannot be determined due to the cross-sectional study design. The irregular treatment schedule may have resulted from conflict-related access restrictions, population displacement, logistical challenges, disruptions to public health services, and the COVID-19 pandemic. However, the cross-sectional design and absence of an annually treated comparison group precluded quantification of the independent effect of the prolonged interval between treatment rounds.

The decline in TF_1–9_ prevalence to below 5% in all districts indicates substantial progress toward meeting the active trachoma component of the criteria for elimination as a public health problem [6]. Accordingly, no additional MDA rounds for active trachoma are currently indicated in the surveyed district; however, strengthened surveillance for recrudescence and continued implementation of F and E components of the SAFE strategy remain essential. Nevertheless, eliminating trachoma as a public health problem also requires reducing the prevalence of TT to below 0.2% among adults aged 15 years or older. Because TT was not assessed in the present study, the findings cannot confirm that the complete elimination criteria have been met. Further assessment of TT among adults is therefore required to determine progress toward achieving all WHO elimination targets. Post-MDA surveillance surveys are required after two years to monitor for potential recrudescence of active trachoma [23, 24]. MDA can substantially lower community transmission by reducing the human reservoir of infection, which is crucial for controlling NTDs such as trachoma. The prevalence observed in the present study is lower than that reported in Ibb (3.1–7.6%) and Hodeidah (2.3–3.1%) in impact surveys conducted in 2019 [10], suggesting a steady decline over time. A higher prevalence of 10.9% was also reported among primary schoolchildren aged 7–12 years in Bajil district of Hodeidah in 2020, and a similarly higher prevalence was reported among schoolchildren aged 6–12 years in Sana’a city in 2021 [11, 12]. The substantial decline in prevalence observed in the present study is comparable to the progress reported in several other countries [14, 24, 25].

In the present study, neither the sex nor the age of children was significantly associated with TF_1–9_. This finding is inconsistent with those reported among children aged 1–9 years in Papua New Guinea and Zimbabwe [26, 27], where younger age was identified as an independent predictor of TF. This difference may reflect variations in transmission patterns, population characteristics, or environmental conditions. However, only a small number of TF_1–9_ cases were detected in the present study, which may have reduced the statistical power to detect modest differences according to age or sex. Therefore, the absence of statistically significant associations should not be interpreted as evidence that children’s age and sex have no influence on TF_1–9_ risk.

TF_1–9_ in the present study was significantly higher among children living in households headed by individuals older than 40 years in the univariable analysis; however, the variable was not retained as an independently associated factor in the final multivariable model. This finding suggests that the age of the household head may have acted as a proxy for other household-level socioeconomic, sanitation, or hygiene-related factors rather than exerting an independent effect. In addition, the small number of TF-positive cases may have reduced the statistical power of the multivariable model to identify an adjusted association. Further studies with larger numbers of TF-positive cases are needed to clarify the household-level factors underlying this observed association, including the trachoma-related knowledge and hygiene practices of household heads. Previous research has shown that inadequate knowledge of trachoma among household heads was significantly associated with increased odds of TF_1–9_ [15]. Several studies conducted in other settings have demonstrated that larger household sizes independently predict TF among children [28–31], possibly because close contact and overcrowding facilitate the transmission of ocular and nasal secretions. However, no statistically significant association between household size and TF_1–9_ was observed in the present study, and the limited number of TF_1–9_ cases should again be considered when interpreting this absence of association. This finding aligns with that reported among Kenyan children [32], suggesting that the dynamics of trachoma transmission may vary across contexts and with differences in hygiene practices.

Regarding WASH conditions, unimproved sanitation and visible facial discharge were associated with TF₁–₉ in the exploratory multivariable model; however, the wide CIs indicate substantial imprecision and warrant cautious interpretation. These findings are consistent with broader evidence linking active trachoma to inadequate sanitation and poor facial cleanliness. A systematic review and meta-analysis reported lower odds of active trachoma among children with access to sanitation, facial cleanliness, absence of ocular or nasal discharge, regular face washing, and soap use [33]. In Yemen, facial uncleanliness was associated with active trachoma, while the absence of household latrines and the accumulation of solid waste were more common in highly affected governorates [34]. Similar associations with inadequate sanitation have been reported in many endemic settings. For example, the absence of a household latrine independently predicted inflammatory trachoma in rural Egypt [35], latrine use was associated with a lower risk of trachoma in rural Tanzania with no significant difference between shared and private facilities [36], and inadequate household sanitation was associated with active trachoma in northeastern Nigeria and Burundi [37, 38]. The independent association between visible facial discharge and TF_1–9_ in the present study is consistent with findings from eastern Ethiopia, where an unclean face was independently associated with trachoma among schoolchildren [29], while ocular discharge, nasal discharge, and flies on the face were independently associated with active trachoma among schoolchildren in southern Ethiopia [39]. Facial uncleanliness, particularly the presence of nasal or ocular discharge, may facilitate transmission through direct contact and by attracting eye-seeking flies that mechanically transfer infected secretions between children [40, 41]. Longitudinal evidence further supports the potential importance of sanitation in reducing trachoma transmission. For instance, in Ethiopian communities receiving azithromycin MDA, each 10% increase in community latrine use was associated with an approximately 2% reduction in ocular *C. trachomatis* prevalence during the subsequent year [42]. Similarly, an Ethiopian systematic review and meta-analysis found higher odds of active trachoma among members of households lacking toilet facilities or access to improved water sources [43]. Conversely, low residual active trachoma prevalence in Oman was observed in the context of very high levels of facial cleanliness and widespread access to piped or tanker-supplied water [44], underscoring the potential contribution of sustained facial cleanliness and environmental improvement measures to trachoma control.

Several limitations should be considered when interpreting these findings. While significant associations were observed for selected risk factors, cautious interpretation is warranted because the low positivity rate makes estimates vulnerable to sparse data bias, separation and model overfitting. In particular, only 11 children were identified as TF-positive, which limited the statistical power of the multivariable binary logistic regression analysis and resulted in wide CIs around the adjusted estimates. The statistically significant findings may also be susceptible to sparse-data bias and should not be interpreted as precise effect estimates or definitive evidence of independent prediction. Instead, they should be regarded as exploratory associations that identify potential contributors to residual TF transmission. Because facial cleanliness was assessed by direct visual observation without photographic grading, observer variability and potential misclassification cannot be excluded. Accordingly, the significant association between facial cleanliness and TF_1–9_ should be interpreted cautiously. Similarly, regular hand and face washing with soap and water was self-reported and not observationally verified, making this measure susceptible to recall and social desirability bias, which may have attenuated the observed association with TF_1–9_. In addition, the same ophthalmologists assessed facial cleanliness immediately before grading the children for TF. Although they did not administer the household questionnaire or receive the completed forms, they were not formally masked to all household-level information, creating a plausible risk of information bias, particularly when assessing subjective or borderline clinical findings.

Although the included districts achieved mean programmatic MDA coverage exceeding 80% across the three rounds, the observational cross-sectional design limits the extent to which the observed reduction in TF_1–9_ prevalence can be attributed to MDA alone. The study could not distinguish MDA’s independent contribution from other possible influences. A further sampling-related limitation is that only one child was randomly selected from each household containing multiple eligible children. Although this approach ensured equal household representation and reduced within-household correlation, it may have limited the detection of within-household clustering of TF and underestimated the burden in households with multiple affected children. Future surveys should consider examining all children aged 1–9 years within selected households, with appropriate adjustment for household-level clustering. Furthermore, operational and logistical constraints, including difficulties accessing some communities, population mobility, and the limited survey period, prevented the consistent documentation of non-participating households and reasons for non-participation, thereby limiting the ability to assess potential selection bias. Additionally, the study did not account for all confounding variables, particularly environmental and socioeconomic factors, which may influence predictors of infection and could lead to spurious associations or obscure true associations. It is noteworthy that both the 2013 and 2024 surveys were conducted using the WHO-recommended two-stage cluster sampling framework and by trained and certified ophthalmologists, making comparison between the two surveys methodologically reasonable. However, because the 2013 survey was conducted before the conflict that emerged in March 2015, whereas the 2024 survey was conducted in the context of displacement and a prolonged humanitarian crisis, potential changes in the demographic, socioeconomic, and WASH characteristics of the sampled population cannot be excluded. Conflict-related population movement may have altered the composition of households remaining in the study districts, introducing possible selection bias. Therefore, the observed decline in TF prevalence should be interpreted with caution as a programmatic reduction over time rather than as definitive evidence of the independent effect of MDA alone. Finally, because this study did not estimate TT prevalence among adults aged ≥ 15 years, it cannot confirm elimination of trachoma as a public health problem. Despite the limitations and caution required in interpreting the results, the study provides useful programmatic evidence of the low prevalence of active trachoma following three rounds of MDA and identifies household sanitation and facial cleanliness as potential areas for continued intervention and investigation.

## Conclusions

Following three rounds of MDA, the marked reduction in TF_1–9_ prevalence in previously highly endemic districts of Ibb and Hodeidah governorates below the WHO threshold of 5% represents substantial progress toward achieving the active trachoma component of elimination as a public health problem. While further MDA is not currently indicated for active trachoma in the surveyed districts, TT assessment and continued post-MDA surveillance remain necessary before drawing conclusions about the full elimination status. Further attention should also be given to districts showing comparatively smaller reductions, particularly Al Qanawis. Poor household sanitation and unclean facial status, due to nasal or ocular discharge, could independently predict TF_1–9_; however, these findings should be interpreted with caution given the small number of TF-positive children. Therefore, strengthened sanitation and facial cleanliness interventions under the SAFE strategy are crucial for preventing recrudescence and sustaining progress toward elimination. Programmatic priorities should include targeted hygiene education, promotion of regular face washing, improved access to and use of sanitation facilities, and coordination between the national trachoma program and WASH services in communities where residual transmission risks persist.

## Data Availability

Yes, I have research data.

## Abbreviations

AOR: Adjusted odds ratio
CI: Confidence interval
COVID-19: Coronavirus disease 2019
EU: Evaluation unit
IQR: Interquartile range
MDA: Mass drug administration
NTD: Neglected tropical disease
OR: Odds ratio
SAFE: Surgery, Antibiotics, Facial Cleanliness and Environmental Improvements
SD: Standard deviation
SPSS: Statistical Package for the Social Sciences
TF_1–9_: Trachomatous inflammation–follicular among children aged 1–9 years
TF: Trachomatous inflammation–follicular
TT: Trachomatous trichiasis
WASH: Water, sanitation, and hygiene
WHO: World Health Organization
